# A methylome-wide mQTL analysis reveals associations of methylation sites with *GAD1* and *HDAC3* SNPs and a general psychiatric risk score

**DOI:** 10.1038/tp.2016.275

**Published:** 2017-01-17

**Authors:** D M Ciuculete, A E Boström, S Voisin, H Philipps, O E Titova, M Bandstein, L Nikontovic, M J Williams, J Mwinyi, H B Schiöth

**Affiliations:** 1 Division of Functional Pharmacology, Department of Neuroscience, Biomedicinskt Centrum, Uppsala University, Uppsala, Sweden

## Abstract

Genome-wide association studies have identified a number of single-nucleotide polymorphisms (SNPs) that are associated with psychiatric diseases. Increasing body of evidence suggests a complex connection of SNPs and the transcriptional and epigenetic regulation of gene expression, which is poorly understood. In the current study, we investigated the interplay between genetic risk variants, shifts in methylation and mRNA levels in whole blood from 223 adolescents distinguished by a risk for developing psychiatric disorders. We analyzed 37 SNPs previously associated with psychiatric diseases in relation to genome-wide DNA methylation levels using linear models, with Bonferroni correction and adjusting for cell-type composition. Associations between DNA methylation, mRNA levels and psychiatric disease risk evaluated by the Development and Well-Being Assessment (DAWBA) score were identified by robust linear models, Pearson’s correlations and binary regression models. We detected five SNPs (in *HCRTR1*, *GAD1*, *HADC3* and *FKBP5*) that were associated with eight CpG sites, validating five of these SNP–CpG pairs. Three of these CpG sites, that is, cg01089319 (*GAD1*), cg01089249 (*GAD1*) and cg24137543 (*DIAPH1*), manifest in significant gene expression changes and overlap with active regulatory regions in chromatin states of brain tissues. Importantly, methylation levels at cg01089319 were associated with the DAWBA score in the discovery group. These results show how distinct SNPs linked with psychiatric diseases are associated with epigenetic shifts with relevance for gene expression. Our findings give a novel insight on how genetic variants may modulate risks for the development of psychiatric diseases.

## Introduction

Genome-wide association studies have shown that the level of DNA methylation, probably the best-studied epigenetic mark, is partly associated with nearby single-nucleotide polymorphisms (SNPs).^1, 2, 3^ An increasing number of SNPs have been associated with the pathogenesis of psychiatric disorders.^4^ In this context, genetic variants in various genes, such as *COMT*, *BDNF*, *GAD1* or *APOE*,^5, 6, 7, 8^ are repeatedly highlighted. However, the exact mechanisms behind the interplay between methylation levels and polymorphisms are poorly understood. A further elucidation of the interaction between genetic and epigenetic mechanisms may allow a better understanding of the mechanistic role of genetic polymorphisms in development of complex psychiatric diseases.

Brain maturation occurs during adolescence through processes such as synaptic pruning.^9^ Normal brain development and function are highly dependent on DNA methylation as stable chemical modification of cytosines in CpG dinucleotides. The importance of these reactions was demonstrated for several cognitive processes, such as learning and memory^10^ and neuronal activity.^11^ This includes a previous study that showed a relationship between differential DNA methylation and genes involved in γ-aminobutyric acid (GABA)-ergic pathways.^11^ In 2013, Domschke *et al.*^12^ revealed that, compared with healthy controls, *GAD1* methylation levels were lower in adults with panic disorder. Interestingly, a previous study showed that individuals homozygous for the major allele of rs4680 (within *COMT*) showed stress-related methylation changes, which was not observed in heterozygotes.^13^ Higher levels of DNA methylation repress transcription by inhibiting the binding of transcription factors or by changing microRNA expression.^14^ These findings indicate that changes in DNA methylation may contribute to the compensation and/or modulation of interindividual genetic variation^15^ and may be able to induce alterations in the complex regulatory landscape of gene expression levels in psychiatric disorders.

For we believe the first time, our study specifically investigates to what extent 37 psychiatric disease-related SNPs are connected to methylation changes (methylation quantitative trait loci (mQTL) analyses) in 223 adolescent individuals showing a differently strong risk for the development of psychiatric illnesses, as evaluated by the Development and Well-Being Assessment (DAWBA) band score. In this context, we further scrutinize whether differentially methylated regions associated with genetic variations influence gene expression, as well as to what extent they modulate the risk for disease development.

## Materials and methods

### Subjects

#### Discovery data set

The present study included a total of 130 adolescent volunteers aged between 14 and 16, recruited between November 2012 and January 2013. The included subjects were randomly selected from public school in Uppsala County, with the aim to investigate potential psychiatric risk factors in youth. Self-reported information regarding basic physiological parameters, for example, height, age and medication, was provided by the participants. In addition, body weight was measured for body mass index calculation. This data set served as discovery data set in our study.

#### Replication data set

In the replication stage, 93 samples from the same study, but characterized later in the time frame 2013–2014, were evaluated. The selection criteria included individuals with a risk for psychiatric diagnoses higher than 15%, aged between 14 and 16 years. The same parameters as in the discovery group were recorded for these individuals. Both studies were approved by the Regional Ethics Committee in Uppsala, and all participants gave their written informed consent.

#### Expression data set

Eleven healthy male volunteers aged between 18 and 40 years were recruited from the region of Uppsala, Sweden, between 2013 and 2014. Blood analyses were performed before and after a meal intake. The analyses regarding methylation and expression profiles were corrected for proportions of different cell types. More details about this healthy group as well as about preprocessing of the methylation specimens and expression patterns in this cohort can be found elsewhere.^16^

### Phenotype assessment

The risk for psychiatric diseases was assessed by performing a DAWBA web-based interview designed for individuals in the age range 5–17 years to generate Diagnostic and Statistical Manual of Mental Disorders (DSM-IV) and International Classification of Diseases (ICD)-10-based psychiatric diagnoses. DAWBA consists of two versions of individual standardized questionnaires, administrated to adolescents and their parents. Computer-generated diagnostic predictions were only used in the present study. The average of the ‘probability bands’ was computer-assisted generated referring to several diagnoses such as anxiety disorders, depression, post-traumatic stress disorder, autism, separation anxiety disorder and obsessive compulsive disorder. DAWBA diagnoses are given in the range of less than 0.1% to over 70% probability that the individual could experience one of the mentioned DSM-IV or ICD-10-based diagnosis. A more detailed description of DAWBA-level bands and details about validation steps are given in Goodman *et al.*^17^

### Genotyping and DNA methylation

All study participants were genotyped using the Illumina Golden Gate array at the SNP&SEQ SciLife Platform at Uppsala University. On the basis of the literature search regarding associations of risk variants with psychiatric disorders, that is, eating disorders, panic disorder, obsessive-compulsive disorder, major depression and bipolar disorder,^18^ and on their inclusion in the microarray-assisted genotyping approach, we investigated a set of 37 SNPs with minor allele frequency higher than 5%. All SNPs were checked for Hardy–Weinberg equilibrium using *χ*^2^-test. SNPs were considered in disequilibrium if *P*<0.01. Each SNP was coded as 0 (homozygous major allele), 1 (heterozygous genotype) or 2 (homozygous minor allele). A dominant model was assumed for the SNPs. Genome-wide DNA methylation analysis was carried out using the Illumina Infinium HumanMethylation450 BeadChip (Illumina, San Diego, CA, USA, 450k). Description of experimental procedures can be found in Supplementary File 1.

### DNA methylation preprocessing

**Figure 1:**
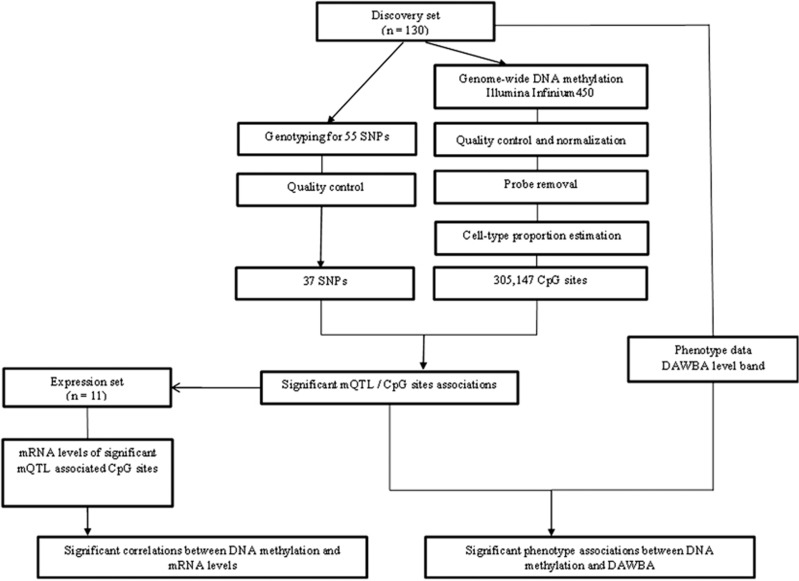
Workflow of the study. DAWBA, Development and Well-Being Assessment; mQTL, methylation quantitative trait loci; SNP, single-nucleotide polymorphism.

Preprocessing of methylation data included background correction, probe exclusion, adjustment of type I and type II probes and removal of batch effects. The robust pipeline also included outlier detection by principal component analyses and white blood cell correction (Supplementary File 2). These analysis steps were performed using the lumi, sva, limma, wateRmelon and FactoMineR packages available in Bioconductor and operable in the R version 3.1.3 software (R is freely available under the GNU General Public License). The experimental flow of the discovery set is presented in Figure 1.

### Criteria of sample exclusion

Samples were excluded based on the following criteria: samples that were shown to be outliers in any of the five quality-control plots generated by MethylAid^19^ (rotated M versus U plot, overall sample-dependent control plot, bisulfite conversion control plot, overall sample-independent control plot and detection *P*-value plot) without changing the default thresholds (0 sample) and samples that were outliers for the first two principal components (1 sample). The procedure was repeated in the same manner for the validation cohort where none of the samples were excluded.

### Evaluation of DNA methylation sites and mQTLs in brain and blood

To investigate to what extent epigenetic shifts in whole blood are of functional relevance in the brain, we correlated chromatin marks in brain and blood. On the basis of the availability of chromatin state data derived using Hidden Markov Models (HMMs), the following eight brain tissues were analyzed: brain angular gyrus, brain anterior caudate, brain cingulate gyrus, brain hippocampus, brain inferior temporal lobe, brain substantia nigra and peripheral blood mononuclear primary cells. Data were loaded from Roadmap Epigenomics Project of 37/hg19 version of human genome in the WashU Epigenome Browser. For easier visualization, the 18-state model for the production of the segmentations according to gene-regulatory role was reduced to five regions, indicating the functionality by different colors: red, for active/flanking active/bivalent/poised transcription start site (TSS); yellow, for active/bivalent/genic enhancer; orange, for flanking bivalent TSS/enhancer; green, for active transcription; and grey, for repressed polyComb state. Using this reliable tool, information about regions representing chromatin states overlapping significant CpG sites and mQTL was obtained. In addition, potential regulatory effects of the CpG sites on multiple genes and the specificity of the association with mQTLs were considered by examining long-range interactions. The long-range interaction mapping was derived using chromatin analysis by paired-end tag sequencing libraries from the ENCODE consortium. Four different cell lines, that is, erythrocytic leukaemia cells (K562), breast cancer (MCF-7), cervical cancer (HelaS3) and human colon carcinoma (HCT-116), targeting the transcription factors RNA polymerase II and CCCTC-binding factor (CTCF), an insulator protein with diverse functions,^20^ were used. Data were downloaded from the WashU Epigenome Browser, 37/hg19 version.

### Ubiquitous, tissue-specific and cell type-specific *in vivo* transcribed enhancers

In order to get information about the *in vivo* relevance of the CpG sites for expression, we used data produced by the FANTOM5 project. Ubiquitous, tissue-specific (brain, blood) and cell type-specific (T cells, neurons) *in vivo* enhancers as defined by CAGE tags were downloaded from the Transcribed Enhancer Atlas website.^21^

### Linkage disequilibrium of mQTLs

Linkage disequilibrium (LD) data were obtained using SNAP Proxy web tool,^22^ with Northern Europeans from Utah (CEU) as the population selection. Two SNPs having *r*^2^=1 and *D*′=1 were considered in perfect LD.

### Statistical analysis

Beta values of methylation were used for graphical illustration. For statistical analysis, we transformed the beta values to *M*-values, which have been shown to be statistically more robust.^23^ Statistical analyses were performed using Bioconductor, R (version 3.1.3) and SPSS software (version 22; SPSS, Armonk, NY, USA). To guarantee the reliability and power of data analyses, a confirmatory approach was chosen, investigating two independent cohorts with regard to SNP–methylation interaction.

#### Linear models

The association between SNPs and DNA methylation was tested through linear models using the *limma* R package, suitable for large-scale methylation studies,^16, 24^ applying an empirical Bayes method based on a moderated *t*-statistic. We assumed a linear model where the *M*-values of each individual CpG site i were used as the quantitative dependent trait and categorical variables, for example, genotype (*G*) and sex (*S*), together with continuous variables, for example, age (*A*), body mass index, PC1 and PC2, were included as covariates. The coefficient *b*_i_ represents the strength of association between methylation levels and variable of interest and *ε*_i_ refers to the unexplained variability.


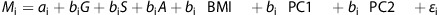


All analyses were adjusted for multiple testing using the Bonferroni correction. Adjusted two-sided *P*-value<0.05 was considered significant. A known limitation of the epigenome-wide association analyses is an inflated number of false positives.^25, 26^ Therefore, the genomic inflation factor (*λ*) for all SNP–CpG analyses was calculated using the estlambda function of *GenABEL* R package.^27^ The analyses were restricted to 500 kb up- and downstream of each SNP. The targeted analyses were performed applying a likelihood ratio test, with lrtest function of the *lmtest* package.^28^ Here, to correct for false positives, two-sided *q*-values were calculated using the *qvalue* package^29^ and values <0.05 were considered significant.

#### Binary regression models

Significant CpG sites were tested in relationship with the general DAWBA band. Binominal tests were applied between DAWBA score outcome and continuous *M*-values of the individual CpG site and adjusting for body mass index, age, sex and DAWBA version, that is, score generated based on adolescent questionnaire or both adolescent and parent questionnaires. For these analyses, DAWBA scores were ranked into two categories (0 and 1). Individuals from the discovery data set with a DAWBA risk score below 50% were defined as ‘Low risk’ (category 0; 87.4%) and included the levels 0 (<0.1%), 1 (≈0.5%), 2 (≈3%) and 3 (≈15%) of DAWBA. The individuals with levels 4 (≈50%) and 5 (>70%), having a risk higher than 50%, were assigned to the ‘High risk’ category (12.6%). The same subgroups were built in the replication set. According to the DAWBA version, 37.2% of individuals from the discovery set and 60.2% from the replication set had the DAWBA-level band generated based only on adolescent questionnaire. Separate analyses including only the complete DAWBA general band score, based on both adolescent and parent questionnaires, were performed for the discovery and replication data sets, adjusting for body mass index, sex and age. Two-tailed *P*-values<0.05 were considered significant.

#### Robust linear regression models and Pearson’s correlations

Validated DNA methylation changes were tested in association with the gene expression levels, computing robust linear regression models and Pearson’s correlations in R environment. Robust linear models are recommended to be used in case of a small sample size, to account for any outliers or heteroscedasticity in the data.^30^ The *robust* package was used for these computations. The sated state of the 11 individuals was chosen to perform these analyses because of the similarity with the discovery and replication data sets. The transcripts corresponding to significant CpG loci were determined from the original Illumina annotation referring to the nearest gene to each probe or by a potential regulatory effect on other genes as described in the literature. Two-sided *P*-values <0.05 were considered significant.

## Results

### Study data sets

The outcome of demographic and clinical variables of the discovery and replication set is illustrated in Table 1. The discovery data set was retrieved from 129 adolescents, who were in the majority female subjects. The mean age was 15.33±0.60 years. There were no substantial demographic differences between the discovery and replication groups. The subjects from both cohorts were categorized into two categories according to the level band score of DAWBA, that is, in the ‘Low risk’ (*n*=113, respectively, *n*=44) and ‘High risk’ group (*n*=16, respectively, *n*=49). The DAWBA general band was used as the outcome variable in the analyses, whereas separate symptoms were not analyzed, given the small number of cases in the discovery cohort (Supplementary Table 1A).

### Effect of cell heterogeneity across samples

**Figure 2:**
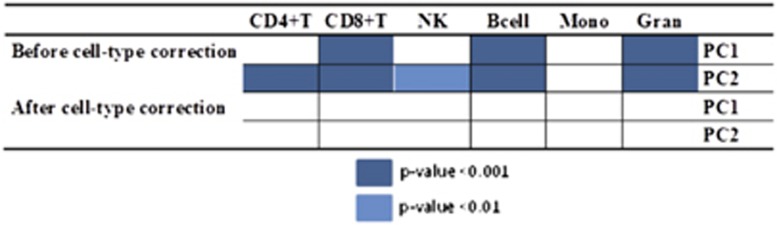
Illustration of cell-type composition effect using principal component analysis (PCA). The heatmap indicates significant correlations between the first two principal components and the estimations of blood cell types. After cell-type correction, no association between the first two principal components and cell type are longer observed. Bcell, B cell; Gran, granulocyte; Mono, monocytes; NK, natural killer; PC, principal component.

Our mQTL analyses were adjusted for cell-type proportions detected in whole blood. To test the reliability of the correction for blood cell proportions, we calculated the first two principal components for unadjusted beta values. Subsequently, we performed Pearson’s correlation analyses between the first two principal components and cell-type estimations, that is, CD4+T cells, CD8+T cells, B cells, NK, Mono and Gran, before and after adjustment (Figure 2). The importance of cell-type correction is proven by evaluating the percentage of explained variance. The heatmap shown in Figure 2 illustrates that the cell-type correction accounted for cell heterogeneity. The first and second principal components (PC1 and PC2), calculated based on the unadjusted beta values, explained 29% variance, whereas the same first two principal components applied to beta values after blood cell estimation accounted for 8%.

### Relationship between genotype and methylation profiles

The study workflow is illustrated in Figure 1. After having performed the necessary preprocessing steps for the Illumina 450k array data, 305 147 CpG loci were investigated for their association with 37 SNPs, which were earlier associated with psychiatric diseases. Analyses were performed based on the data of 129 individuals from the discovery group after one outlier was excluded. Top hit associations and related unadjusted *P*-values of all mQTL are shown in Supplementary Table 2. We detected a significant association for eight SNP–CpG pairs (*P*_bonf_<0.05, corresponding to *P*<1.6 × 10^−7^), that is, a significant relationship between the level of DNA methylation at a distinct CpG site and a polymorphism (Table 2). These eight pairs consisted of five SNPs (13.5% of tested SNPs) and seven CpG sites (0.002% of tested CpG sites). The SNPs included rs10914453, rs2058725, rs2241165, rs2530223 and rs9296158 and are located in or nearby genes previously related to psychiatric disorders, that is, *HCRTR1*, *GAD1*, *HDAC3* and *FKBP5*. The SNP rs2241165 is in perfect LD with the *GAD1* variant rs2270335. The closest genes to the detected significant CpG sites included *PEF1*, *GAD1*, *C11orf9*, *DIAPH1* and *PCDHGC3*.

A highly significant positive association was found for the methylation site cg01089319 (*GAD1* gene) with two genetic variants of the gene *GAD1* (rs2058725 (*P*=0.0003) and rs2241165 (*P*=2.61e−06)). In addition, cg01089249 showed a strong relationship to the variant rs2241165 (*GAD1*, *P*=1.44e−02). The CpG site represented by cg24137543 was significantly associated with rs2530223 within *HDAC3* (*P*=0.009). All significant identified methylation sites on the genome-wide scale were validated using targeted analyses, except in case of cg18766608, which was not located in the range of 500 kb up- or downstream of rs2058725 (data not shown).

We sought to increase the power of our analyses by confirming our findings in a replication set comprising of 93 individuals. During this step the limma models were restricted to five genetic variants significantly associated with DNA methylation levels in the discovery set. After correction for multiple testing, five SNP–CpG pairs were validated, including the SNPs rs10914453 (*HCRTR1*), rs2058725 (*GAD1*), rs2241165 (*GAD1*) and rs2530223 (*HDAC3*). Four of seven CpG loci were confirmed to have associations with the genetic variants in the same direction as in the discovery data set. All associations with a *P*_bonf_<0.05, corresponding to *P*<1.58 × 10^−7^, were considered significant. In line with the results obtained in the discovery data set, the CpG site cg01089319 was significantly associated with the *GAD1* SNPs rs2058725 (*P*=5.89e−07) and rs2241165 (*P*=5.98e−06). The other top two associations detected in the discovery group were also confirmed in the replication group (cg24137543 and rs2530223 (*P*=7.59e−06); cg01089249 and rs2241165 (*P*=1.33e−06; Table 2).

**Figure 3:**
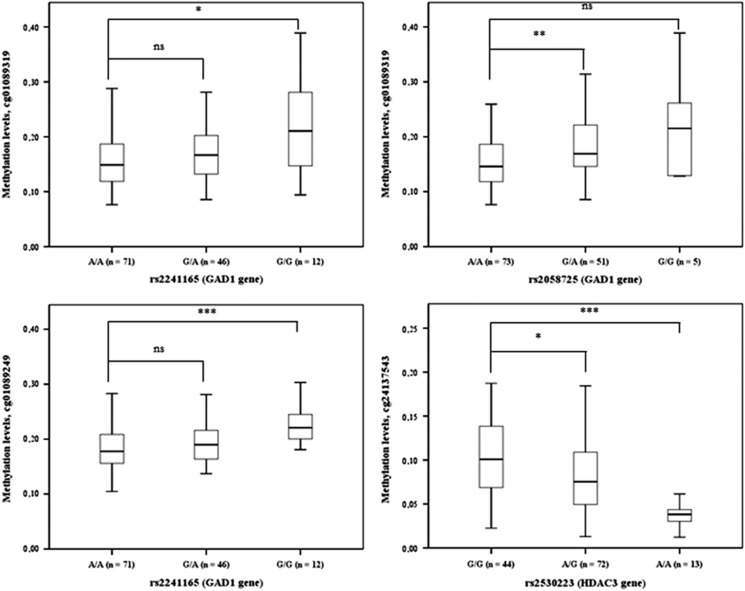
Associations between genotype data at the four validated SNPs and methylation levels at three unique CpG sites (cg01089319, cg01089249 and cg24137543) in the discovery set. Distribution of the beta values at the methylation sites is illustrated for individuals carrying zero, one and two minor alleles. *P-value<0.05 (Student’s *t*-test); **P-value<0.01 (Student’s *t*-test); ***P-value<0.001 (Student’s *t*-test). SNP, single-nucleotide polymorphism.

Given our hypothesis that a subset of SNPs are associated with methylation levels, DNA methylation levels were stratified according to the number of minor alleles in the discovery data set (Figure 3). Carriers of the minor allele (G allele) of *GAD1* SNPs rs2058725 and rs2241165 showed higher methylation levels at cg01089319 and cg01089249. Conversely, carriers of the ancestral allele at rs2530223 showed higher methylation levels. In the discovery cohort, the inflation factor was below 1, except for one variant at rs2530223 (*λ*=1.09). For the replication set all *λ* values were below 1.

### Genomic context of the CpG sites significantly associated with *GAD1* and *HDAC3* mQTLs

**Figure 4:**
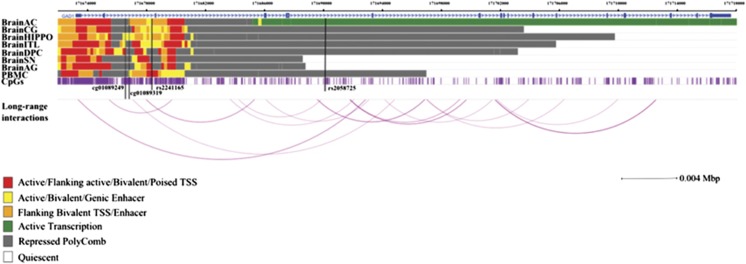
Genomic context of the most significant CpG sites associated with SNPs rs2241165 and rs2058725. Genomic positions of RefSeq genes are displayed in the top part, indicated by blue arrows. The positions of the significant CpG sites are highlighted by black lines. For the investigation of specificity of the associations, long-range interactions were derived from four cell lines targeting two transcription factors. Associations are represented by arcs. Only long-range interactions containing significant CpGs were illustrated. The intensity of the arc is proportional to the strength of the interaction between the two regions. As analyses were performed based on data obtained in blood, chromatin marks overlapping in brain and blood cells were investigated. Chromatin states of eight tissues downloaded from the 37/hg19 WashU Epigenome Browser are illustrated. Each functional role of a segment is indicated by a particular color. BrainAC, brain anterior caudate; BrainAG, brain angular gyrus; BrainCG, brain cingulate gyrus; BrainDPC, brain dorsolateral prefrontal cortex; BrainHIPPO, brain hippocampus; BrainITL, brain inferior temporal lobe; BrainSN, brain substantia nigra; PBMC, peripheral blood mononuclear primary cells; SNP, single-nucleotide polymorphism; TSS, transcription start site.

**Figure 5:**
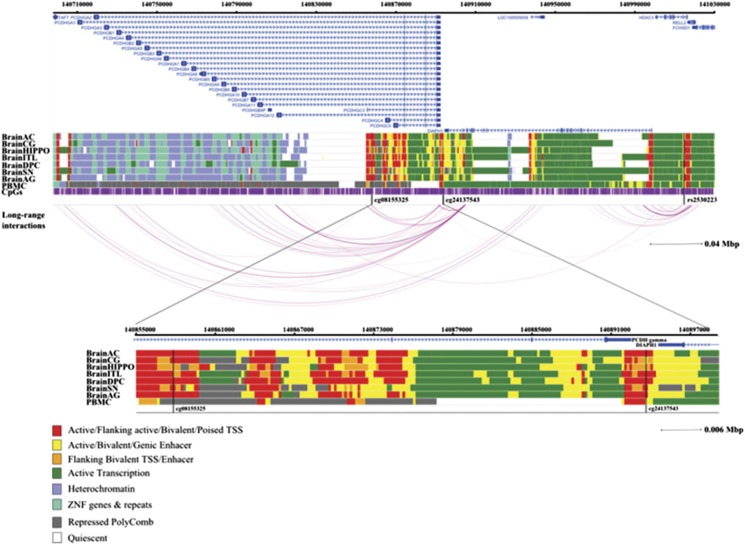
Genomic context of the most significant CpG sites (cg08155325 and cg24137543) associated with SNP rs2530223. Genomic positions of RefSeq genes are displayed in the top part, indicated by blue arrows. The positions of the significant CpG sites are highlighted by black lines. For the investigation of the potential regulatory effect of the significant CpG sites on other genes and of the specificity of the associations, long-range interactions were derived from four cell lines targeting two transcription factors. Associations are represented by arcs. Only long-range interactions containing significant CpGs are illustrated. The intensity of the arc is proportional to the strength of the interaction between the two regions. As analyses were performed based on data obtained in blood, chromatin marks overlapping in brain and blood cells were investigated. Chromatin states of eight tissues downloaded from the 37/hg19 WashU Epigenome Browser are illustrated. Each functional role of a segment is indicated by a particular color. BrainAC, brain anterior caudate; BrainAG, brain angular gyrus; BrainCG, brain cingulate gyrus; BrainDPC, brain dorsolateral prefrontal cortex; BrainHIPPO, brain hippocampus; BrainITL, brain inferior temporal lobe; BrainSN, brain substantia nigra; PBMC, peripheral blood mononuclear primary cells; SNP, single-nucleotide polymorphism; TSS, transcription start site.

The four CpG sites significantly associated with *GAD1* and *HDAC3* SNPs were further investigated with regard to the tissue specificity of the association and their potential functional implication in gene regulation. Therefore, we compared the functional role of these CpG sites in different brain tissues and blood cells with regard to gene regulatory relevance and/or in interaction with nearby genes, offering a broad landscape between chromatin states and potential regulatory activity. All four CpG sites show interaction arcs, with mQTLs indicating that the associations may not be tissue-specific. The cg01089319 site (*GAD1*) did not show relevant long-range interactions with other genes (data not shown). Instead, it is located within an enhancer region of functional relevance in the hippocampus and peripheral blood mononuclear primary cell, according to the results obtained with the tool chromHMM (Figure 4). The second CpG site in *GAD1* (cg01089249) is also located in an enhancer region, but is only detectable in the hippocampus. The CpG site cg24137543 associated with rs2530223 (*HDAC3*) is located within important gene regulatory regions, that is a TSS, an enhancer or flanking active TSS/enhancer throughout all investigated brain tissues and peripheral blood mononuclear primary cell. Interestingly, this CpG site is located in or show long interactions with the TSS or enhancer regions of γ-protocadherins (γ*-Pcdh*) subfamily genes (Figure 5).

### Relationship between methylation changes at significant and validated CpG sites and gene expression

The genomic context of validated CpG sites (cg00112260, cg01089319, cg01089249 and cg24137543) led to a more detailed analysis regarding their role in gene transcription (Table 3). We tested associations between CpG loci from *GAD1* (cg01089319 and cg01089249) and expression levels of *GAD1*, *DNMT1*, *DNMT3a* (ref. 31) and *COMT* (ref. 5). We also evaluated the relationship between methylation changes at cg24137543 and the expression of *HDAC3* and γ*-Pcdh* subfamily. After computing robust linear regression models, three significant associations between the methylation level at cg01089319 and *GAD1* expression (*P*=0.03, coefficient=−1.14), as well as the methylation level at cg24137543 and both *HDAC3* and *PCDHGA6* expression levels (*P*=1.25e−06, coefficient=1.49 and respectively, *P*=0.014, coefficient=−1.44) were identified. Using Pearson’s correlation analysis, the latter association between methylation levels at cg24137543 and *PCDHGA6* expression was validated (*P*=0.04, correlation coefficient (cor)=−0.60). Furthermore, an additional negative correlation between cg01089249 (*GAD1*) and *COMT* expression (*P*=0.04, cor=−0.61) was detected. No correlations were identified between methylation levels at cg00112260 and the expression of the *HCRTR1* associated mQTL (Table 3).

### Relationship between methylation levels at validated CpGs and DAWBA risk scores

We tested the association between methylation changes of significant CpG loci and DAWBA score in subsequent binary regression analyses initially in the discovery cohort. The methylation of CpG site cg01089319 was found to be strongly and significantly associated with two categories of the DAWBA general score, that is, ‘Low risk’ and ‘High risk’ score (*P*=0.031, odds ratio=3.03, 95% confidence interval 1.10–8.32). The separate analysis supported the association between methylation levels at cg01089319 and the complete DAWBA general bands (*P*=0.033). The differences in methylation levels at cg01089319 are 3% between carriers and non-carriers of rs2058725 and rs2241165, respectively. These findings were not confirmed in the validation set, which has a more homogeneous composition (Supplementary Table 1B).

## Discussion

To our knowledge, this is the first study that investigates the association between SNPs known to be related to different psychiatric diseases and the genome-wide methylation pattern. By further investigating the association of SNP-related CpG sites with gene expression and phenotypic psychiatric disease measures, this paper elucidates novel mechanistic insights on how the detected CpG sites in focus may influence the expression of genes hypothesized to have a pathogenetic role in psychiatric diseases in adolescents.

We detected that the methylation state of eight CpG sites was associated with SNPs and replicated five of these pairs in whole blood, after cell-type confounding and strict Bonferroni correction. These significant variants include the following genes *HCRTR1*, *GAD1*, *HDAC3* and *FKBP5,* earlier associated with neuropsychiatric diseases.^32^ Interestingly, the *GAD1* SNP rs2241165 is in perfect LD with rs2270335, which has been previously linked with grey matter loss in childhood-onset schizophrenia.^33^ Moreover, we extended our analyses on the relationship of the methylation levels with gene expression, identifying associations with GAD1 and COMT expression. Importantly, one of our identified CpG site (cg01089319) within the *GAD1* gene is significantly hypermethylated in the individuals with higher general psychiatric risk (DAWBA) score in discovery data set. These findings point that these SNPs are partly reflecting epigenetic regulatory loops, changing the expression of important psychiatric susceptibility genes.

Previous studies revealed that epigenetic shifts appear to be especially of importance for the regulation of pathways involved in neuronal development.^34, 35, 36, 37, 38^ Indeed, in our study on adolescent individuals, we found five psychiatric risk variants to be associated with DNA methylation. One of the most striking finding is the role of these methylation shifts in *GAD1* gene expression. GAD1 is a critical enzyme for the synthesis of GABA, the most important inhibitory neurotransmitter in the brain. Here we show that the two GAD1 variants are significantly associated with DNA methylation of the CpG sites cg01089319 and cg0108924, detecting a difference of 3% between carriers and non-carriers of the minor allele. Importantly, chromatin states map the CpG loci in an enhancer region in the hippocampus (Figure 4), an observation that is consistent with the role of *GAD1* expression in the hippocampus.^39^

Alterations in GABA activity have been shown to be a result of lower *GAD1* expression induced by epigenetic mechanisms.^40, 41^ Lower GABA concentrations were found in patients with mood disorder, bipolar disorder or depression compared with controls.^42, 43, 44^ Another gene, which might regulate GABA neuronal excitability, is *COMT* (ref. 45). This enzyme is involved in dopamine inactivation. The exact mechanism of how this neurotransmitter regulates GABAergic activity is, however, unclear. On the basis of our results, we support the hypothesis of a potential functional interaction of *COMT* and *GAD1* in the GABA circuit.^5, 46^ We identified a significant inverse correlation between methylation levels at cg01089249 (*GAD1*) and *COMT* expression. However, we did not observe a change in *GAD1* messenger RNA (mRNA) expression. This could be because of the fact that *GAD1* undergoes important post-transcriptional modification.^47^ The lack of correlations between methylation levels and mRNA profiles can be attributed to the effect of methylation changes on alternative splicing^48^ or to an unspecific signal as multiple transcripts are recognized by one probe.^49^

The small difference in DNA methylation at cg01089319 observed is significantly associated with the level of DAWBA general band. Our regression model accounted for 20% of the variation of the risk level measured by DAWBA in the discovery set. The methylation levels at this CpG site are associated with two variants within the *GAD1* gene, suggesting an additional mechanism of how these SNPs affect the risk for different psychiatric disorders. This finding allows the hypothesis that genetic variants and associated methylation changes are not disorder-specific but associated to several psychiatric disorders.^50^ Importantly, the discovery cohort is a population-based cohort, whereas the replication cohort has a more homogenous composition with regard to DAWBA scores. This might be a possible reason that this association was not validated.

Another variant that was associated with differences in DNA methylation profiles was the exonic SNP rs2530223, within the gene *HDAC3*. This gene is strongly expressed in the hippocampus.^51^ The enzyme HDAC3 was shown to have a role in normal brain development^52^ and specifically in long-term memory.^53^ Moreover, this protein represses the transcription factor GATA-2 (ref. 54), which modulates GABAergic neurons in the midbrain.^55^ The associated CpG site cg24137543 is located in sequence areas of regulatory importance throughout all brain regions and blood cells according to chromatin states mapping and shows interaction arcs with the mQTL (Figure 4). This observation suggests that the detected association may not be tissue-specific. Furthermore, methylation at cg24137543 was inversely correlated with *PCDHGA6* gene expression. This association supports our previously identified interaction with transcription factor sites in γ-Pcdh subfamily genes from chromatin analysis by paired-end tag sequencing libraries. γ-protocadherin genes were shown to be expressed in synapses^56^ and deficiency of γ-Pcdh transcripts differentially influences GABAergic neurons in mice.^57^ The cg24137543 location within an *in vivo* enhancer region of neurons strengthens the evidence for a potential modulatory effect on γ-Pcdh transcripts in neurons.

The strength of our study is the genome-wide approach, revealing the most relevant changes of the CpG sites. Consistently, we could validate the associations of the identified CpG sites with distinct SNPs by independent local analyses. We specifically investigated 37 SNPs known to be strongly associated with psychiatric diseases. It cannot be excluded that additional SNPs may show similar association patterns with methylation. Although we accounted for different cell-type proportions in blood, the preferred tissue for biomarker analysis, additional studies in brain tissue would provide valuable information about additional tissue-specific SNP/CpG site associations and connected expression changes, which are not reflected in blood. Furthermore, it will be of value to validate the CpG/mQTL associations in larger cohorts, taking changes of the transcriptional expression of associated genes into account. Finally, to increase the power of the analysis on the association between methylation levels and the DAWBA general bands, we combined scores generated exclusively by adolescent questionnaires with scores generated based on both adolescent and parent questionnaires. As the adolescent questionnaire does not cover all behavioral and hyperactivity-type disorders^17^ present in the parent questionnaire, this heterogeneity in our primary outcome variable could bias downstream results. However, we adjust for this potential bias by controlling for this difference in our analysis. In addition, the separate analysis including only the complete DAWBA general band score, based on both adolescent and parent questionnaires, confirmed the positive association between level of methylation and DAWBA score. We were specifically interested in analyzing SNP–methylation interactions in individuals at risk for psychiatric diseases in adolescence, encompassing multiple psychiatric diseases and neurobiological changes. Future studies of larger sample size are needed to further investigate the associations in specific diseases, such as, for example, depression, general anxiety and obsessive-compulsive disorder.

In conclusion, we succeeded to define an epigenetic landscape that associates with genetic markers related to psychiatric diseases and gene expression. Our findings show that the effect of several important psychiatric disease-related SNPs appears at least partly to be the result of associated epigenetic shifts that lead to alterations in GABAergic signaling in the human brain.

## Figures and Tables

**Table 1:** Discovery and replication data sets’ description

	*Discovery set (*n=*129)*			*Replication set (*n=*93)*		
	*Low risk*	*High risk*	P*-value*^a^	*Low risk*	*High risk*	P*-value*^a^
Sex (male) *n* (%)	33 (25.6)	4 (3.1)	NS	14 (15.0)	5 (5.3)	**0.019**
Age (years)±s.d.	15.34±0.59	15.25±0.68	NS	15.77±0.61	15.70±0.64	NS
BMI (kg/m^2^)±s.d.	21.37±2.89	24.68±6.3	NS	22.23±3.16	22.06±3.32	NS

Abbreviations: BMI, body mass index; DAWBA, Development and Well-Being Assessment.

Continuous variables are shown as mean±s.d.

Individuals with a general DAWBA psychiatric risk score below 50% were defined as ‘Low risk’ and included 0 (<0.1%), 1 (≈0.5%), 2 (≈3%) and 3 (≈15%) level bands of the DAWBA score. Individuals with level bands 4 (≈50%) and 5 (>70%), having a risk higher than 50%, were assigned to the ‘High risk’ category.

a Two-tailed analysis tests the difference between the ‘Low risk’ and ‘High risk’ group using the Student’s *t*-test for continuous variables and the *χ*^2^-test for categorical variables. Bold value signifies *P*-values<0.05.

**Table 2:** Genome-wide significant psychiatric-associated CpG sites

					*Discovery Set (*n*=129)*					*Replication Set (*n*=93)*				
					*% DNA methylation (s.d.)*					*% DNA methylation (s.d.)*				
*Significant CpGs*	*Distance to closest TSS (bp)*	*Closest TSS gene*	*Associated SNP (gene)*	*Distance to SNP (bp)*	*Non-carriers*^a^	*Carriers*^b^	*Log fold change*	*Unadj.* P*-value*	*Adjusted* P*-value (Bonf.)*	*Non-carriers*^a^	*Carriers*^b^	*Log fold change*	*Unadj.* P*-value*	*Adjusted* P*-value (Bonf.)*
cg00112260	5298	*PEF1*	rs10914453 (*HCRTR1)*	29 885	81 (0.5)	78 (0.4)	−0.44	4.72e−08	**0.01**	82 (0.03)	78 (0.03)	−0.40	3.68e−08	**1.15e−02**
cg01089319	3610	*GAD1*	rs2058725 (*GAD1*)	13 311	15 (0.04)	18 (0.06)	0.55	1.00e−09	**0.0003**	15 (0.03)	22 (0.05)	0.65	1.87e−12	**5.89e−07**
cg01089319	3610	*GAD1*	rs2241165 (*GAD1*)	1569	15 (0.04)	18 (0.06)	0.60	8.57e−12	**2.61e−06**	15 (0.03)	22 (0.05)	0.64	1.90e−11	**5.98e−06**
cg01089249	3354	*GAD1*	rs2241165 (*GAD1*)	1825	18 (0.04)	19 (0.03)	0.32	4.72e−08	**1.44e−02**	18 (0.01)	22 (0.03)	0.34	4.24e−12	**1.33e−06**
cg24137543	12 394	*DIAPH1*	rs2530223 (*HDAC3*)	120 860	10 (0.04)	7 (0.04)	−0.95	3.24e−08	**0.009**	11 (0.03)	6 (0.02)	−1.05	2.41e−11	**7.59e−06**
cg08155325	2245	*PCDHGC3*	rs2530223 (*HDAC3*)	156 681	71 (0.09)	62 (0.09)	−0.67	1.86e−08	**0.005**	68 (0.08)	60 (0.06)	—	1.62e−07	NS
cg02569698	518	*TULP1*	rs9296158 (*FKBP*5)	86 953	20 (0.04)	16 (0.03)	−0.41	8.91e−08	**0.02**	17 (0.02)	14 (0.02)	—	9.72e−05	NS
cg18766608	−31 764	*C11orf9*	rs2058725 (*GAD1*)	110 201 764	94 (0.01)	92 (0.01)	-0.32	1.41e−07	**0.04**	—	—	—	NS	NS

Abbreviations: bp, base pair; Bonf, Bonferroni corrected; NS, nonsignificant; PC, principal component; SNP, single-nucleotide polymorphism; TSS, transcription start site; Unadj., unadjusted.

The table shows the associations that remain significant after the Bonferroni correction. All 37 investigated SNPs and the top hit associated CpG using unadjusted analyses are shown in Supplementary Table 2.

Associations between SNPs and DNA methylation in the discovery cohort were performed genome-wide using linear models (*limma* package, R). In the replication data set, analyses were restricted to the significantly associated SNPs and CpGs. Covariates included were age, sex, BMI, PC1 and PC2. Shown are the raw and Bonferroni *P*-values.

a Non-carriers of the coding allele.

b Carriers of the coding allele. Bold values signifiy *P*-values<0.05.

**Table 3:** Associations of the significant and validated CpG sites and gene expressions in 11 adult healthy individuals

*CpG site*	*Affymetrix transcript ID*^a^	*Gene expression*	*Coef.*^b^	P*-value*^b^	*Coef.*^c^	P*-value*^c^
cg01089319	NM_000817	*GAD1*	**−**1.14	**0.03**	**—**	NS
	NM_001130823, NM_001379	*DNMT1*	**—-**	NS	**—**	NS
	NM_022552, NM_153759	*DNMT3A*	**—**	NS	**—**	NS
	NM_144589	*COMT*	**—**	NS	**—**	NS
cg01089249	NM_000817	*GAD1*	**—**	NS	**—**	NS
	NM_001130823, NM_001379	*DNMT1*	**—**	NS	**—**	NS
	NM_022552, NM_153759	*DNMT3A*	**—**	NS	**—**	NS
	NM_144589	*COMT*	**—**	NS	**−**0.61	**0.04**
cg24137543	NM_003883	*HDAC3*	1.49	**1.25e−06**	**—**	NS
	NM_018919	*PCDHGA6*	**−**1.44	**0.01**	**−**0.60	**0.04**
cg00112260	NM_001525	*HCRTR1*	**—**	NS	**—**	NS

Abbreviations: Coef, coefficient; NS, nonsignificant.

a According to GeneChip Human Gene 2.1 ST Array annotation file.

b Correlations between methylation levels at CpG sites (*M*-values) and gene expression levels were performed using robust linear regression models. Shown are *P*-values and coefficients.

c Correlations between methylation levels at CpG sites (*M*-values) and gene expression levels are performed using Pearson’s correlation analyses. Shown are *P*-values and coefficients. Bold values signify *P*-values<0.05.

## References

[bib1] Ziller MJ, Gu H, Muller F, Donaghey J, Tsai LT, Kohlbacher O et al. Charting a dynamic DNA methylation landscape of the human genome. Nature 2013; 500: 477–481. 23925113 10.1038/nature12433 PMC3821869

[bib2] Gamazon ER, Badner JA, Cheng L, Zhang C, Zhang D, Cox NJ et al. Enrichment of cis-regulatory gene expression SNPs and methylation quantitative trait loci among bipolar disorder susceptibility variants. Mol Psychiatry 2013; 18: 340–346. 22212596 10.1038/mp.2011.174 PMC3601550

[bib3] Voisin S, Almen MS, Zheleznyakova GY, Lundberg L, Zarei S, Castillo S et al. Many obesity-associated SNPs strongly associate with DNA methylation changes at proximal promoters and enhancers. Genome Med 2015; 7: 103. 26449484 10.1186/s13073-015-0225-4 PMC4599317

[bib4] Sullivan PF, Daly MJ, O’Donovan M. Genetic architectures of psychiatric disorders: the emerging picture and its implications. Nature Reviews Genetics 2012; 13: 537–551. 10.1038/nrg3240 PMC4110909 22777127

[bib5] Straub RE, Lipska BK, Egan MF, Goldberg TE, Callicott JH, Mayhew MB et al. Allelic variation in GAD1 (GAD67) is associated with schizophrenia and influences cortical function and gene expression. Mol Psychiatry 2007; 12: 854–869. 17767149 10.1038/sj.mp.4001988

[bib6] Guerreiro RJ, Gustafson DR, Hardy J. The genetic architecture of Alzheimer's disease: beyond APP, PSENs and APOE. Neurobiol Aging 2012; 33: 437–456. 20594621 10.1016/j.neurobiolaging.2010.03.025 PMC2980860

[bib7] Autry AE, Monteggia LM. Brain-derived neurotrophic factor and neuropsychiatric disorders. Pharmacol Rev 2012; 64: 238–258. 22407616 10.1124/pr.111.005108 PMC3310485

[bib8] Egan MF, Goldberg TE, Kolachana BS, Callicott JH, Mazzanti CM, Straub RE et al. Effect of COMT Val108/158 Met genotype on frontal lobe function and risk for schizophrenia. Proc Natl Acad Sci USA 2001; 98(12): 6917–6922. 11381111 10.1073/pnas.111134598 PMC34453

[bib9] Selemon LD. A role for synaptic plasticity in the adolescent development of executive function. Transl Psychiatry 2013; 3: e238. 23462989 10.1038/tp.2013.7 PMC3625918

[bib10] Day JJ, Sweatt JD. DNA methylation and memory formation. Nat Neurosci 2010; 13: 1319–1323. 20975755 10.1038/nn.2666 PMC3130618

[bib11] Ma DK, Jang MH, Guo JU, Kitabatake Y, Chang ML, Pow-Anpongkul N et al. Neuronal activity-induced Gadd45b promotes epigenetic DNA demethylation and adult neurogenesis. Science 2009; 323: 1074–1077. 19119186 10.1126/science.1166859 PMC2726986

[bib12] Domschke K, Tidow N, Schrempf M, Schwarte K, Klauke B, Reif A et al. Epigenetic signature of panic disorder: a role of glutamate decarboxylase 1 (GAD1) DNA hypomethylation? Progr Neuro-psychopharmacol Biol Psychiatry 2013; 46: 189–196. 10.1016/j.pnpbp.2013.07.014 23906988

[bib13] Ursini G, Bollati V, Fazio L, Porcelli A, Iacovelli L, Catalani A et al. Stress-related methylation of the catechol-O-methyltransferase Val 158 allele predicts human prefrontal cognition and activity. J Neurosci 2011; 31: 6692–6698. 21543598 10.1523/JNEUROSCI.6631-10.2011 PMC6632869

[bib14] Han L, Witmer PDW, Casey E, Valle D, Sukumar S. DNA methylation regulates microRNA expression. Cancer Biol Ther 2007; 6: 1290–1294. 10.4161/cbt.6.8.4486 17660710

[bib15] Ursini G, Cavalleri T, Fazio L, Angrisano T, Iacovelli L, Porcelli A et al. BDNF rs6265 methylation and genotype interact on risk for schizophrenia. Epigenetics 2016; 11: 11–23. 26889735 10.1080/15592294.2015.1117736 PMC4846123

[bib16] Rask-Andersen M, Bringeland N, Nilsson EK, Bandstein M, Olaya Bucaro M, Vogel H et al. Postprandial alterations in whole-blood DNA methylation are mediated by changes in white blood cell composition. Am J Clin Nutr 2016; 104: 518–525. 27385611 10.3945/ajcn.115.122366

[bib17] Goodman A, Heiervang E, Collishaw S, Goodman R. The 'DAWBA bands' as an ordered-categorical measure of child mental health: description and validation in British and Norwegian samples. Soc Psychiatr Psychiatr Epidemiol 2011; 46: 521–532. 10.1007/s00127-010-0219-x 20376427

[bib18] Gratacos M, Costas J, de Cid R, Bayes M, Gonzalez JR, Baca-Garcia E et al. Identification of new putative susceptibility genes for several psychiatric disorders by association analysis of regulatory and non-synonymous SNPs of 306 genes involved in neurotransmission and neurodevelopment. Ame J Med Genet B 2009; 150B: 808–816. 10.1002/ajmg.b.30902 19086053

[bib19] van Iterson M, Tobi EW, Slieker RC, den Hollander W, Luijk R, Slagboom PE et al. MethylAid: visual and interactive quality control of large Illumina 450k datasets. Bioinformatics 2014; 30: 3435–3437. 25147358 10.1093/bioinformatics/btu566

[bib20] Phillips JE, Corces VG. CTCF: master weaver of the genome. Cell 137: 1194–1211. 10.1016/j.cell.2009.06.001 PMC3040116 19563753

[bib21] Andersson R, Gebhard C, Miguel-Escalada I, Hoof I, Bornholdt J, Boyd M et al. An atlas of active enhancers across human cell types and tissues. Nature 2014; 507: 455–461. 24670763 10.1038/nature12787 PMC5215096

[bib22] Machiela MJ, Chanock SJ. LDlink: a web-based application for exploring population-specific haplotype structure and linking correlated alleles of possible functional variants. Bioinformatics 2015; 31: 3555–3557. 26139635 10.1093/bioinformatics/btv402 PMC4626747

[bib23] Du P, Zhang X, Huang C-C, Jafari N, Kibbe WA, Hou L et al. Comparison of Beta-value and M-value methods for quantifying methylation levels by microarray analysis. BMC Bioinformatics 2010; 11: 1–9. 21118553 10.1186/1471-2105-11-587 PMC3012676

[bib24] Smyth GK. Linear models and empirical bayes methods for assessing differential expression in microarray experiments. Stat Appl Genet Mol Biol 2004; 3, 3. 10.2202/1544-6115.1027 16646809

[bib25] Barfield RT, Almli LM, Kilaru V, Smith AK, Mercer KB, Duncan R et al. Accounting for population stratification in DNA methylation studies. Genet Epidemiol 2014; 38: 231–241. 24478250 10.1002/gepi.21789 PMC4090102

[bib26] Zou J, Lippert C, Heckerman D, Aryee M, Listgarten J. Epigenome-wide association studies without the need for cell-type composition. Nat Method 2014; 11: 309–311. 10.1038/nmeth.2815 24464286

[bib27] developers Gp. Genome-wide SNP association analysis 2015. Available from https://cran.r-project.org/web/packages/GenABEL/GenABEL.pdf.

[bib28] Achim Z, Torsten H. Diagnostic checking in regression relationships. R News 2002; 2: 7–10.

[bib29] Bass JDSwcfAJ DA, Robinson D. qvalue: Q-value estimation for false discovery rate control 2015. Available from http://github.com/jdstorey/qvalue.

[bib30] Joubert BR, Haberg SE, Nilsen RM, Wang X, Vollset SE, Murphy SK et al. 450K epigenome-wide scan identifies differential DNA methylation in newborns related to maternal smoking during pregnancy. Environ Health Perspect 2012; 120: 1425–1431. 22851337 10.1289/ehp.1205412 PMC3491949

[bib31] Costa E, Chen Y, Dong E, Grayson DR, Kundakovic M, Maloku E et al. GABAergic promoter hypermethylation as a model to study the neurochemistry of schizophrenia vulnerability. Exp Rev Neurother 2009; 9: 87–98. 10.1586/14737175.9.1.87 19102671

[bib32] Hettema JM, An SS, Neale MC, Bukszar J, van den Oord EJCG, Kendler KS et al. Association between glutamic acid decarboxylase genes and anxiety disorders, major depression, and neuroticism. Mol Psychiatry 2006; 11: 752–762. 16718280 10.1038/sj.mp.4001845

[bib33] Addington AM, Gornick M, Duckworth J, Sporn A, Gogtay N, Bobb A et al. GAD1 (2q31.1), which encodes glutamic acid decarboxylase (GAD67), is associated with childhood-onset schizophrenia and cortical gray matter volume loss. Mol Psychiatry 2005; 10: 581–588. 15505639 10.1038/sj.mp.4001599

[bib34] Jakovcevski M, Akbarian S. Epigenetic mechanisms in neurological disease. Nat Med 2012; 18: 1194–1204. 22869198 10.1038/nm.2828 PMC3596876

[bib35] Lister R, Mukamel EA, Nery JR, Urich M, Puddifoot CA, Johnson ND et al. Global epigenomic reconfiguration during mammalian brain development. Science 2013; 341: 1237905. 23828890 10.1126/science.1237905 PMC3785061

[bib36] Numata S, Ye T, Hyde TM, Guitart-Navarro X, Tao R, Wininger M et al. DNA methylation signatures in development and aging of the human prefrontal cortex. Am J Hum Genet 2012; 90: 260–272. 22305529 10.1016/j.ajhg.2011.12.020 PMC3276664

[bib37] Grayson DR, Guidotti A. The dynamics of DNA methylation in schizophrenia and related psychiatric disorders. Neuropsychopharmacology 2013; 38: 138–166. 22948975 10.1038/npp.2012.125 PMC3521968

[bib38] Mill J, Tang T, Kaminsky Z, Khare T, Yazdanpanah S, Bouchard L et al. Epigenomic profiling reveals DNA-methylation changes associated with major psychosis. Am J Hum Genet 2008; 82: 696–711. 18319075 10.1016/j.ajhg.2008.01.008 PMC2427301

[bib39] Heckers S, Stone D, Walsh J, Shick J, Koul P, Benes FM. Differential hippocampal expression of glutamic acid decarboxylase 65 and 67 messenger RNA in bipolar disorder and schizophrenia. Arch Gen Psychiatry 2002; 59: 521–529. 12044194 10.1001/archpsyc.59.6.521

[bib40] Guidotti A, Auta J, Davis JM, Dong E, Grayson DR, Veldic M et al. GABAergic dysfunction in schizophrenia: new treatment strategies on the horizon. Psychopharmacology 2005; 180: 191–205. 15864560 10.1007/s00213-005-2212-8

[bib41] Kundakovic M, Chen Y, Costa E, Grayson DR. DNA methyltransferase inhibitors coordinately induce expression of the human reelin and glutamic acid decarboxylase 67 genes. Mol Pharmacol 2007; 71: 644–653. 17065238 10.1124/mol.106.030635

[bib42] Petty F, Kramer GL, Dunnam D, Rush AJ. Plasma GABA in mood disorders. Psychopharmacol Bull 1990; 26: 157–161. 2236451

[bib43] Guidotti A, Auta J, Davis JM, Di-Giorgi-Gerevini V, Dwivedi Y, Grayson DR et al. Decrease in reelin and glutamic acid decarboxylase67 (GAD67) expression in schizophrenia and bipolar disorder: a postmortem brain study. Arch Gen Psychiatry 2000; 57: 1061–1069. 11074872 10.1001/archpsyc.57.11.1061

[bib44] Hasler G, van der Veen JW, Tumonis T, Meyers N, Shen J, Drevets WC. Reduced prefrontal glutamate/glutamine and gamma-aminobutyric acid levels in major depression determined using proton magnetic resonance spectroscopy. Arch Gen Psychiatry 2007; 64: 193–200. 17283286 10.1001/archpsyc.64.2.193

[bib45] Seamans JK, Gorelova N, Durstewitz D, Yang CR. Bidirectional dopamine modulation of GABAergic inhibition in prefrontal cortical pyramidal neurons. J Neurosci 2001; 21: 3628–3638. 11331392 10.1523/JNEUROSCI.21-10-03628.2001 PMC6762481

[bib46] Marenco S, Savostyanova AA, van der Veen JW, Geramita M, Stern A, Barnett AS et al. Genetic modulation of GABA levels in the anterior cingulate cortex by GAD1 and COMT. Neuropsychopharmacology 2010; 35: 1708–1717. 20357758 10.1038/npp.2010.35 PMC2891897

[bib47] Martin DL, Rimvall K. Regulation of gamma-aminobutyric acid synthesis in the brain. J Neurochem 1993; 60: 395–407. 8419527 10.1111/j.1471-4159.1993.tb03165.x

[bib48] Lev Maor G, Yearim A, Ast G. The alternative role of DNA methylation in splicing regulation. Trends Genet 31: 274–280. 25837375 10.1016/j.tig.2015.03.002

[bib49] Schioth HB, Bostrom A, Murphy SK, Erhart W, Hampe J, Moylan C et al. A targeted analysis reveals relevant shifts in the methylation and transcription of genes responsible for bile acid homeostasis and drug metabolism in non-alcoholic fatty liver disease. BMC Genomics 2016; 17: 462. 27301979 10.1186/s12864-016-2814-z PMC4908840

[bib50] Lee SH, Ripke S, Neale BM, Faraone SV, Purcell SM, Perlis RH et al. Genetic relationship between five psychiatric disorders estimated from genome-wide SNPs. Nat Genet 2013; 45: 984–994. 23933821 10.1038/ng.2711 PMC3800159

[bib51] Broide RS, Redwine JM, Aftahi N, Young W, Bloom FE, Winrow CJ. Distribution of histone deacetylases 1-11 in the rat brain. J Mol Neurosci 2007; 31: 47–58. 17416969 10.1007/BF02686117

[bib52] Norwood J, Franklin JM, Sharma D, D’Mello SR. Histone deacetylase 3 is necessary for proper brain development. J Biol Chem 2014; 289: 34569–34582. 25339172 10.1074/jbc.M114.576397 PMC4263864

[bib53] McQuown SC, Barrett RM, Matheos DP, Post RJ, Rogge GA, Alenghat T et al. HDAC3 is a critical negative regulator of long-term memory formation. J Neurosci 2011; 31: 764–774. 21228185 10.1523/JNEUROSCI.5052-10.2011 PMC3160172

[bib54] Ozawa Y, Towatari M, Tsuzuki S, Hayakawa F, Maeda T, Miyata Y et al. Histone deacetylase 3 associates with and represses the transcription factor GATA-2. Blood 2001; 98: 2116–2123. 11567998 10.1182/blood.v98.7.2116

[bib55] Kala K, Haugas M, Lillevali K, Guimera J, Wurst W, Salminen M et al. Gata2 is a tissue-specific post-mitotic selector gene for midbrain GABAergic neurons. Development 2009; 136: 253–262. 19088086 10.1242/dev.029900

[bib56] Frank M, Ebert M, Shan W, Phillips GR, Arndt K, Colman DR et al. Differential expression of individual gamma-protocadherins during mouse brain development. Mol Cell Neurosci 2005; 29: 603–616. 15964765 10.1016/j.mcn.2005.05.001

[bib57] Chen WV, Alvarez FJ, Lefebvre JL, Friedman B, Nwakeze C, Geiman E et al. Functional significance of isoform diversification in the protocadherin gamma gene cluster. Neuron 2012; 75: 402–409. 22884324 10.1016/j.neuron.2012.06.039 PMC3426296

