# Supplementary material for: A methylome-wide mQTL analysis reveals associations of methylation sites with GAD1 and HDAC3 SNPs and a general psychiatric risk score
Source: Transl Psychiatry. 2017 Jan 17;7(1):e1002–. doi: 10.1038/tp.2016.275 (PMC5545735; doi:10.1038/tp.2016.275)
Supplement: Supplementary Information [file tp2016275x1.docx]

**Supplementary File 1**

**Experimental procedures**

The criteria for the inclusion of SNPs in subsequent analyses were genotype call rates > 90%, low sample heterozygosity (< 3 standard deviations), no duplicated samples and no inheritance errors.

Peripheral blood was collected and used to isolate DNA applying the phenolol-chloroform method. Subsequently, DNA was bisulfite treated using the EZ DNA methylation kit (Zymo Research, Orange, CA). 200 ng of total DNA was used for subsequent genome-wide DNA methylation analysis using the Illumina Infinium HumanMethylation450 BeadChip (Illumina 450k). This array allows the reliable and reproducible interrogation of 485 577 CpG sites associated to 25 953 genes (99% of RefSeq genes). The C or T nucleotides fluorescence signals from the BeadChips were captured and measured using the Illumina iScan scanner. Signal intensity revealed the methylation level of each CpG site and was estimated as β = M (M+U+α), where M and U stand for methylated and unmethylated fluorescence intensities, and α stands for an arbitrary offset applied to stabilize low-intensity β-values.

**Supplementary File 2**

**Annotation**

Besides the original annotation table of the Illumina Infinium HumanMethylation450 BeadChip array, we used also the annotation by Price et *al*. for data interpretation ([1](#_ENREF_1)).

**Background correction, probe exclusion and adjustment of type I and type II probes and removal of batch effects**

In both the discovery and replication data set, background correction and normalization of raw files (.idat files) were performed using the *methylumi* and *asmn* packages of R ([2](#_ENREF_2), [3](#_ENREF_3)). The efficient NOOB method was selected to correct for background artifacts ([4](#_ENREF_4)). 19 374 probes were disqualified from subsequent analysis based on a detection p-value <10^-5^, leaving 466,203 probes for further analysis. Probes on sex chromosomes (which are more difficult to be accurately normalized ([5](#_ENREF_5))), non-specific probes or at annotated SNP loci (as they may only reflect the presence of the genetic polymorphisms ([6](#_ENREF_6))) were also filtered out, which left a total of 305 147 autosomal probes. Different signal distribution and dynamic range may bias the results. Therefore, the Beta Mixture Quantile dilation (BMIQ) normalization (as implemented in *wateRmelon* package ([7](#_ENREF_7))) was applied to remove probe design differences ([8](#_ENREF_8)). Moreover, the use of different analysis plates could introduce an unwanted batch effect. Hence, the ComBat function implemented in the *sva* package of R was used to correct for batch effects ([9](#_ENREF_9)). In the replication set, 314 926 probes passed the quality control and were, thus, included in subsequent analyses.

**White blood cell correction**

The distinct methylation signature of various cell types present in whole blood could be a potential confounder in epigenetic studies ([10](#_ENREF_10)). Therefore, it is critical to correct the analyses with respect to differences in white blood cell composition. We implemented a *minfi*-based statistical procedure of the Houseman algorithm ([11](#_ENREF_11)), which uses raw intensity files to calculate CD4+ and CD8+ T cells, monocytes (Mono), granulocytes (Gran), B cells (Bcell) and natural killer (NK) cells for the discovery and replication data sets.

**Principal component analysis for sample outlier identification and technical variation**

To identify samples outliers and to account for variance across the samples, principal component analysis (PCA) was applied to methylation data using the PCA function implemented in the *FactoMineR* package of R ([12](#_ENREF_12)). Firstly, the most variable CpG sites among all samples were determined within the 95% reference range, calculated as difference between 97.5% and 2.5% percentiles. Using a 95% reference range and a threshold of 0.2, a total of 26 499 CpG sites of the discovery cohort were further used in the calculation of the covariance matrix. Only the first two principal components were taken into consideration, explaining 8% of total variance after cell-type adjustment, since all the other subsequent vectors do not add considerably to the total variance. Outliers were identified with visual inspection of the graphical display of the first two principal components and excluded from further analyses.

In the replication stage, using the 95% reference range and the same threshold, 9 937 hypervariable CpG sites were identified and involved in further analysis. The first two principal components explained 6.7% of total variance after cell-type correction.

|  | Discovery set  (n=129) | | | | | |
| --- | --- | --- | --- | --- | --- | --- |
|  |  |  |  |  |  |  |
|  | <0.1% risk | ≈0.5% risk | ≈3% risk | ≈15% risk | ≈50% risk | >70% risk |
| **Anxiety disorders** |  |  |  |  |  |  |
| Separation anxiety (n) | 72 | 43 | 6 | 5 | 2 | 0 |
| Social fobia (n) | 90 | 21 | 8 | 5 | 5 | 0 |
| Panic disorder (n) | 114 | 11 | 1 | 2 | 1 | 0 |
| Agoraphobia (n) | 114 | 11 | 0 | 2 | 1 | 0 |
| Posttraumatic stress disorder (n) | 119 | 3 | 3 | 3 | 1 | 0 |
| Generalized anxiety disorder (n) | 56 | 56 | 4 | 10 | 3 | 0 |
| **Behavioral disorders** |  |  |  |  |  |  |
| Obsessive-compulsive disorder (n) | 100 | 19 | 5 | 4 | 1 | 0 |
| Conduct disorder (n) | 75 | 37 | 13 | 0 | 3 | 1 |
| **Other disorders** |  |  |  |  |  |  |
| Depression (n) | 76 | 35 | 4 | 12 | 2 | 0 |
| Eating disorder (n) | 49 | 60 | 19 | 0 | 1 | 0 |
| Attention deficit hyperactivity disorder* (n) | 73 | 1 | 2 | 2 | 0 | 0 |
| Oppositional defiant disorder* (n) | 0 | 47 | 30 | 0 | 0 | 1 |
| Tic disorder* (n) | 75 | 2 | 2 | 0 | 0 | 0 |

Table S1A. Distribution of the disease-specific DAWBA scores of the individuals in the discovery set

*Generated only by parent questionnaire

All disorders scores were generated based on both DSM-IV and ICD-10 criteria

|  | Replication set  (n=93) | | | | | |
| --- | --- | --- | --- | --- | --- | --- |
|  |  |  |  |  |  |  |
|  | <0.1% risk | ≈0.5% risk | ≈3% risk | ≈15% risk | ≈50% risk | >70% risk |
| **Anxiety disorders** |  |  |  |  |  |  |
| Separation anxiety (n) | 23 | 49 | 13 | 4 | 0 | 0 |
| Social fobia (n) | 45 | 15 | 9 | 9 | 13 | 0 |
| Panic disorder (n) | 68 | 11 | 1 | 7 | 4 | 0 |
| Agoraphobia (n) | 65 | 22 | 1 | 1 | 2 | 0 |
| Posttraumatic stress disorder (n) | 68 | 6 | 7 | 7 | 3 | 0 |
| Generalized anxiety disorder (n) | 5 | 37 | 7 | 34 | 8 | 0 |
| **Behavioral disorders** |  |  |  |  |  |  |
| Obsessive-compulsive disorder (n) | 43 | 30 | 8 | 7 | 3 | 0 |
| Conduct disorder (n) | 28 | 25 | 22 | 13 | 2 | 1 |
| **Other disorders** |  |  |  |  |  |  |
| Depression (n) | 19 | 25 | 6 | 21 | 14 | 8 |
| Eating disorder (n) | 22 | 32 | 28 | 0 | 9 | 0 |
| Attention deficit hyperactivity disorder* (n) | 26 | 2 | 1 | 2 | 1 | 0 |
| Oppositional defiant disorder* (n) | 0 | 15 | 14 | 0 | 1 | 1 |
| Tic disorder* (n) | 27 | 3 | 1 | 0 | 0 | 1 |

Table S1B. Distribution of the disease-specific DAWBA scores of the individuals in the replication set

*Generated only by parent questionnaire

All disorders scores were generated based on both DSM-IV and ICD-10 criteria

Table S2. Detailed information of the 37 investigated SNPs and the top methylation hit

| Investigated SNP | Chromosome | Coding/other allele | MAF | Top associated CpG site | Unadj. p-value |
| --- | --- | --- | --- | --- | --- |
| rs10914453 | 1 | A/G | 0.31 | cg00112260 | 4.725334e-08 |
| rs3806318 |  | G/A | 0.29 | cg02499768 | 1.033514e-06 |
| rs588387 |  | G/A | 0.36 | cg12078588 | 3.805072e-07 |
| rs1063639 | 2 | A/G | 0.50 | cg25461300 | 1.982034e-07 |
| rs1801262 |  | A/G | 0.39 | cg11728928 | 8.007507e-07 |
| rs2058725 |  | G/A | 0.22 | cg01089319 | 1.002529e-09 |
| rs2241165 |  | G/A | 0.27 | cg01089319 | 8.577570e-12 |
| rs3762556 |  | C/G | 0.31 | cg08618620 | 1.091266e-06 |
| rs769395 |  | G/A | 0.28 | cg04046930 | 1.014131e-06 |
| rs1503433 | 3 | C/A | 0.36 | cg11300098 | 9.645838e-07 |
| rs2245532 |  | G/A | 0.42 | cg03321508 | 1.956871e-06 |
| rs2304725 |  | G/A | 0.29 | cg14240300 | 2.467815e-06 |
| rs7649709 |  | A/C | 0.26 | cg15306012 | 3.282593e-06 |
| rs1868152 |  | A/G | 0.16 | cg15305112 | 1.141431e-05 |
| rs11930311 | 4 | G/C | 0.22 | cg10754596 | 1.041844e-06 |
| rs2530223 | 5 | A/G | 0.37 | cg08155325 | 1.864173e-08 |
| rs9296158 | 6 | A/G | 0.29 | cg02569698 | 8.919316e-08 |
| rs1526083 | 7 | G/A | 0.37 | cg00063471 | 2.850694e-06 |
| rs2692359 |  | G/A | 0.20 | cg11233533 | 1.117168e-05 |
| rs1396860 | 11 | G/A | 0.19 | cg02696670 | 5.600116e-06 |
| rs7938406 |  | G/A | 0.32 | cg04567445 | 1.492288e-06 |
| rs6265 |  | A/G | 0.19 | cg21478902 | 6.741346e-07 |
| rs216250 | 12 | G/A | 0.43 | cg08875431 | 1.247320e-06 |
| rs2289954 |  | A/G | 0.24 | cg01401460 | 7.557496e-07 |
| rs4617664 |  | G/A | 0.37 | cg13569424 | 5.055053e-06 |
| rs557881 |  | A/G | 0.41 | cg09826395 | 1.066488e-05 |
| rs4941807 | 13 | C/A | 0.33 | cg01544270 | 2.887219e-06 |
| rs945032 | 14 | A/G | 0.15 | cg13515881 | 2.258936e-07 |
| rs2376481 | 15 | A/G | 0.47 | cg15067687 | 6.003645e-07 |
| rs3785931 | 17 | G/A | 0.27 | cg00664581 | 3.941246e-06 |
| rs7208505 |  | G/A | 0.39 | cg16414660 | 7.060535e-06 |
| rs2424932 | 20 | A/G | 0.37 | cg08356445 | 6.243327e-07 |
| rs6119954 |  | A/G | 0.18 | cg05017226 | 6.446316e-07 |
| rs6136667 |  | G/C | 0.13 | cg09137301 | 7.510987e-07 |
| rs1555048 | 22 | A/G | 0.29 | cg21600563 | 4.128807e-06 |
| rs4680 |  | G/A | 0.43 | cg23601416 | 3.268175e-06 |
| rs6971 |  | A/G | 0.32 | cg19402939 | 8.812075e-06 |

SNP, single nucleotide polymorphism

MAF, minor allele frequency

**References**

1. Price ME, Cotton AM, Lam LL, Farre P, Emberly E, Brown CJ, et al. Additional annotation enhances potential for biologically-relevant analysis of the Illumina Infinium HumanMethylation450 BeadChip array. Epigenetics & chromatin. 2013;6(1):4. PubMed PMID: 23452981. Pubmed Central PMCID: PMC3740789. Epub 2013/03/05. eng.

2. Sean Davis PD, Sven Bilke, Tim Triche, Jr., Moiz Bootwalla. Handle Illumina methylation data2015. Available from: https://[www.bioconductor.org/packages/3.3/bioc/manuals/methylumi/man/methylumi.pdf](http://www.bioconductor.org/packages/3.3/bioc/manuals/methylumi/man/methylumi.pdf).

3. Anna Decker PY. All sample mean normalization2014. Available from: <http://bioconductor.riken.jp/packages/2.14/bioc/manuals/asmn/man/asmn.pdf>.

4. Triche TJ, Jr., Weisenberger DJ, Van Den Berg D, Laird PW, Siegmund KD. Low-level processing of Illumina Infinium DNA Methylation BeadArrays. Nucleic acids research. 2013 Apr;41(7):e90. PubMed PMID: 23476028. Pubmed Central PMCID: PMC3627582. Epub 2013/03/12. eng.

5. Fortin JP, Labbe A, Lemire M, Zanke BW, Hudson TJ, Fertig EJ, et al. Functional normalization of 450k methylation array data improves replication in large cancer studies. Genome biology. 2014;15(11). PubMed PMID: 25599564.

6. Chen Y-a, Lemire M, Choufani S, Butcher DT, Grafodatskaya D, Zanke BW, et al. Discovery of cross-reactive probes and polymorphic CpGs in the Illumina Infinium HumanMethylation450 microarray. Epigenetics. 2013;8(2):203-9. PubMed PMID: PMC3592906.

7. Schalkwyk LC. wateRmelon: Illumina 450 methylation array normalization and metrics2013. Available from: https://[www.bioconductor.org/packages/release/bioc/html/wateRmelon.html](http://www.bioconductor.org/packages/release/bioc/html/wateRmelon.html).

8. Teschendorff AE, Marabita F, Lechner M, Bartlett T, Tegner J, Gomez-Cabrero D, et al. A beta-mixture quantile normalization method for correcting probe design bias in Illumina Infinium 450 k DNA methylation data. Bioinformatics (Oxford, England). 2013 Jan 15;29(2):189-96. PubMed PMID: 23175756. Pubmed Central PMCID: PMC3546795. Epub 2012/11/24. eng.

9. Johnson WE, Li C, Rabinovic A. Adjusting batch effects in microarray expression data using empirical Bayes methods. Biostatistics (Oxford, England). 2007 Jan;8(1):118-27. PubMed PMID: 16632515. Epub 2006/04/25. eng.

10. Reinius LE, Acevedo N, Joerink M, Pershagen G, Dahlén S-E, Greco D, et al. Differential DNA Methylation in Purified Human Blood Cells: Implications for Cell Lineage and Studies on Disease Susceptibility. PLoS ONE. 2012;7(7):e41361.

11. Aryee MJ, Jaffe AE, Corrada-Bravo H, Ladd-Acosta C, Feinberg AP, Hansen KD, et al. Minfi: a flexible and comprehensive Bioconductor package for the analysis of Infinium DNA methylation microarrays. Bioinformatics (Oxford, England). 2014 May 15;30(10):1363-9. PubMed PMID: 24478339. Pubmed Central PMCID: PMC4016708. Epub 2014/01/31. eng.

12. Lê S, Josse J, Husson F. FactoMineR: An R Package for Multivariate Analysis. 2008. 2008 2008-03-18;25(1):18. Epub 2008-03-18.
